# Direct analysis in real time-mass spectrometry for rapid quantification of five anti-arrhythmic drugs in human serum: application to therapeutic drug monitoring

**DOI:** 10.1038/s41598-020-72490-w

**Published:** 2020-09-23

**Authors:** Yuzhou Gui, Youli Lu, Shuijun Li, Mengqi Zhang, Xiaokun Duan, Charles C. Liu, Jingying Jia, Gangyi Liu

**Affiliations:** 1 Central Laboratory, Shanghai Xuhui Central Hospital, Shanghai, 200031 People’s Republic of China; 2 Zhongshan-Xuhui Hospital, Fudan University, Shanghai, 200031 People’s Republic of China; 3 Shanghai Engineering Research Center of Phase I Clinical Research & Quality Consistency Evaluation for Drugs, Shanghai, 200031 People’s Republic of China; 4 ASPEC Technologies Limited, Beijing, 100101 People’s Republic of China

**Keywords:** Biochemistry, Cardiovascular diseases

## Abstract

Therapeutic drug monitoring (TDM) is necessary for the optimal administration of anti-arrhythmic drugs in the treatment of heart arrhythmia. The present study aimed to develop and validate a direct analysis in real time tandem mass spectrometry (DART–MS/MS) method for the rapid and simultaneous determination of five anti-arrhythmic drugs (metoprolol, diltiazem, amiodarone, propafenone, and verapamil) and one metabolite (5-hydroxy(OH)-propafenone) in human serum. After the addition of isotope-labeled internal standards and protein precipitation with acetonitrile, anti-arrhythmic drugs were ionized by DART in positive mode followed by multiple reaction monitoring (MRM) detection. The use of DART–MS/MS avoided the need for chromatographic separation and allowed rapid and ultrahigh throughput analysis of anti-arrhythmic drugs in a total run time of 30 s per sample. The DART–MS/MS method yielded satisfactory linearity (R^2^ ≥ 0.9906), accuracy (86.1–109.9%), and precision (≤ 14.3%) with minimal effect of biological matrixes. The method was successfully applied to analyzing 30 clinical TDM samples. The relative error (RE) of the concentrations obtained by DART–MS/MS and liquid chromatography-tandem mass spectrometry (LC–MS/MS) was within ± 13%. This work highlights the potential usefulness of DART for the rapid quantitative analysis of anti-arrhythmic drugs in human serum and gives rapid feedback in the clinical TDM practices.

## Introduction

Heart arrhythmia, characterized by irregular rhythms of heartbeat, seriously affects over 33 million people worldwide^1^ and also lays a heavy burden upon the health-care systems of many countries. Since there is no cure for this disease, patients have to take anti-arrhythmic drugs for a life-long time^2^. The optimal use of anti-arrhythmic drugs is necessary for the life quality of arrhythmia patients. However, some of the widely used anti-arrhythmic drugs have a narrow therapeutic window and evident inter-individual variability in their pharmacokinetics. Amiodarone has a typical therapeutic plasma concentrations of 500–2,500 ng/mL. Serum concentrations exceeding the therapeutic range may cause adverse reactions like pulmonary fibrosis, as well as various toxicities in cutaneous, neurological, and gastrointestinal systems^3^. Propafenone has an effective plasma concentration from 40 to 3,000 ng/mL. Patients with deficient hepatic cytochrome P450 (CYP) 2D6 function are more susceptible to side-effects of the central nervous system due to marked reduction in hepatic clearance^4^. Other anti-arrhythmic drugs like β-blockers and calcium-channel blockers have definite clinical efficacy, but missing doses happen frequently among patients^5^. Thus, therapeutic drug monitoring (TDM) of anti-arrhythmic drugs is required to avoid serious toxicity and obtain the desired clinical benefit^6^. Our previous work presented a method for the simultaneous determination of ten anti-arrhythmic drugs and one metabolite using liquid chromatography-tandem mass spectrometry (LC–MS/MS)^7^. However, the method, as well as other reported methods, generally requires chromatographic separation before mass spectrometry analysis, which may pose restraint on the assay throughput and the overall response time to the clinical demand^8–13^. Thus, a rapid quantitative method with acceptable accuracy and precision is very needed for the TDM analysis of the anti-arrhythmic drugs.

DART (Direct Analysis in Real Time) is a promising tool for rapid analysis among the existing analytical techniques^14–16^. It significantly decreases the overall analysis time, eliminates the chromatographic separation step, and reduces the use of solvent^17,18^. As an ionization technique compatible with various mass spectrometers, DART works efficiently for rapid qualitative analysis in fields of the pharmaceutical industry, forensic science, and health, materials analysis^19,20^. However, previous applications of DART to quantitative TDM analysis are very limited^21,22^. To the best of our knowledge, two obstacles may affect its use in quantitative analysis. One is the lack of the reproducibility. Like other ambient ionization techniques, DART is liable to be influenced by the changes in temperature, humidity, and sample loading position. The other obstacle is the low throughput sample loading system. As the majority of DART samples are loaded onto a 10 or 12 sample loading system, it is insufficient for clinical TDM samples in large quantities^23^.

In this study, we aimed to develop and validate a simple, rapid and reliable DART tandem mass spectrometry (DART–MS/MS) method for the determination of five anti-arrhythmic drugs and one metabolite in human serum. Stable isotope-labeled analogs were added as an internal standard in an attempt to normalize the instability of ambient ionization. QuickStrip 96 sample card and an XZ transmission module were adopted to expand the total throughput. The method was validated in terms of linearity, selectivity, specificity, accuracy, precision, recoveries, and matrix effect. The validated DART–MS/MS method was applied to the rapid and quantitative analysis of clinical TDM samples.

## Material and methods

### Reagents and chemicals

Standards of anti-arrhythmic drugs (metoprolol, diltiazem, amiodarone, propafenone, 5-hydroxy(OH)-propafenone, verapamil), and stable isotope-labeled compounds (metoprolol-d7, amiodarone-d4, propafenone-d5 and 5OH-propafenone-d5) were supplied by Toronto Research Chemicals (TRC) with purity > 98%. Chemical structures of the analytes were shown in Supplementary Fig. S1 online. HPLC-grade methanol and acetonitrile were purchased from Merck Millipore. Ultra-pure water was prepared with a Milli-Q Plus water system.

### Preparation of solutions

For each analyte, two sets of 1 mg/mL stock solutions were prepared in methanol. One set was used for the preparation of calibration standards, and the other for the preparation of quality control samples. The working solutions of calibration standards and quality controls were produced by diluting stock solution into a series of levels with methanol/water (1:1). Final concentrations of the spiked serum calibration points and the quality control samples for each analyte were presented in Table 1.

**Table 1:** The concentration of calibration standards and quality control samples.

Analyte	Calibration standards (ng/mL)						Quality control (ng/mL)			
	S1	S2	S3	S4	S5	S6	LLOQ	Low	Middle	High
Metoprolol	10	40	200	1,000	2000	4,000	10	80	1,600	3,200
Diltiazem	10	40	200	1,000	2000	4,000	10	80	1,600	3,200
Amiodarone	50	200	1,000	5,000	10,000	20,000	50	400	8,000	16,000
Propafenone	25	100	500	2,500	5,000	10,000	25	200	4,000	8,000
5OH-propafenone	25	100	500	2,500	5,000	10,000	25	200	4,000	8,000
Verapamil	10	40	200	1,000	2000	4,000	10	80	1,600	3,200

Stock solutions of SIL-IS (metoprolol-d7, amiodarone-d4, propafenone-d5, and 5OH-propafenone-d5) were prepared in methanol at concentrations of 1 mg/mL. The final concentrations were 1 µg/mL, 5 µg/mL, 2.5 µg/mL and 2.5 µg/mL for metoprolol-d7, amiodarone-d4, propafenone-d5, and 5OH-propafenone-d5, respectively.

### Sample preparation

Serum samples 50 μL (spiked or clinical TDM samples), internal standards 10 μL, and methanol 100 μL were sequentially added for protein precipitation. The mixture was vortex-mixed for 10-s and followed by centrifugation at 13,000 g for 3 min. Supernatant 2 μL was added on QuickStrip 96 sample card for DART–MS/MS analysis. Blank serum samples and clinical TDM serum samples were obtained from Shanghai Xuhui Central Hospital. The informed consent was obtained from healthy volunteers and patients. The study protocol was approved by the Ethics Committee at the Shanghai Xuhui Central Hospital (Shanghai, China). The use of human serum in this study conforms to the principles outlined in the Declaration of Helsinki^24^.

### DART–MS/MS conditions

**Figure 1:**
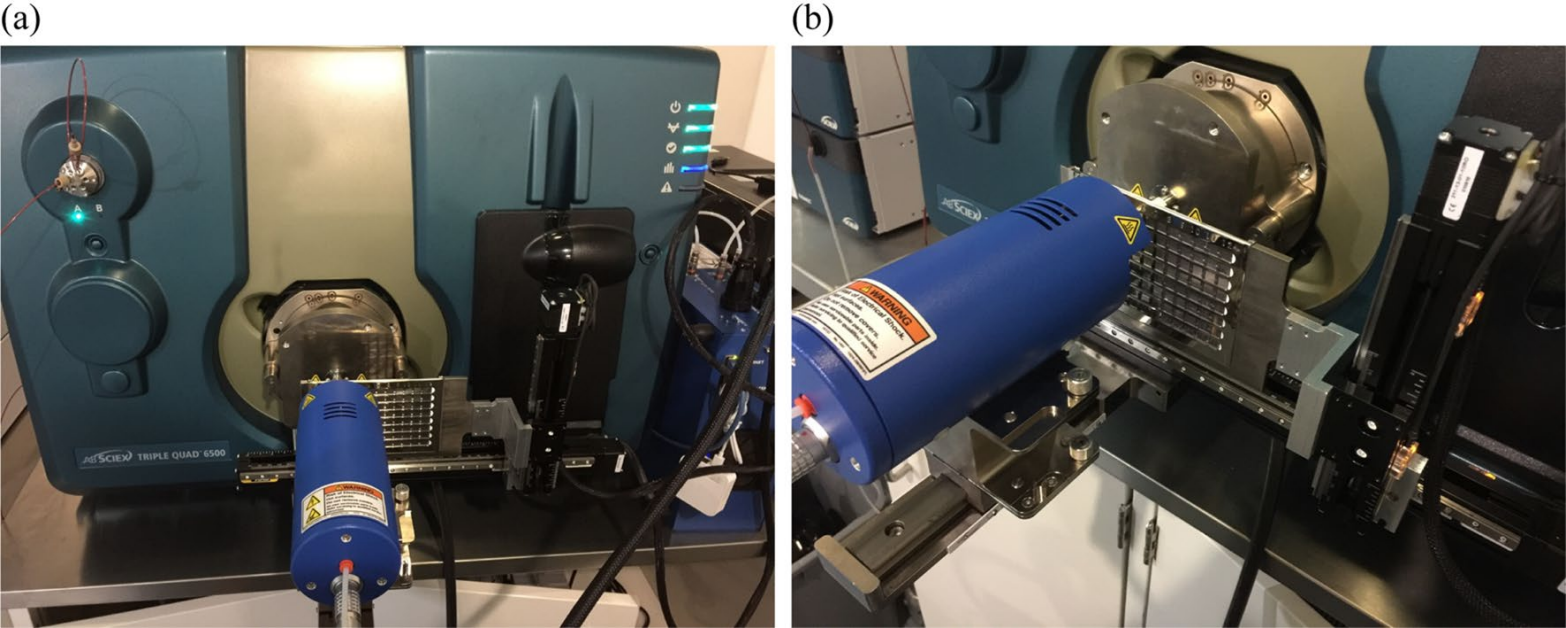
DART coupled with triple quadrupole mass spectrometry. (**a**) Front view; (**b**) side view.

DART (direct analysis in real time) SVP ion source (IonSense, Saugus, MA, USA) was connected to a SCIEX 6500 triple-quadrupole mass spectrometry (SCIEX, Framingham, MA, U.S.A.) for data acquisition. The total acquisition time for each sample was < 30-s. The XZ transmission module and QuickStrip 96 sample card (IonSense Inc., Saugus, MA, USA) with 96 sampling spots were the sample introduction system. As shown in Fig. 1, samples were loaded directly onto the sample card. The sample surface was then controlled automatically by DART software for direct desorption under ambient conditions. The DART ion source was set to the conditions below: positive ionization mode, discharge needle voltage at + 1,500 V, grid electrode voltage at + 350 V. In the run mode, high-purity helium was utilized for desorption and ionization of compounds. In the standby mode, it was replaced by high-purity nitrogen. The pressure of nitrogen and helium was set at 80 psi and the gas temperature was optimized from 200 to 450 °C. X rail moving speed was set from 0.3 to 4 mm/s, with a 15-s delay before the next sample.

The mass spectrum was optimized with 1 μg/mL chemical working solutions on Turbo V Ionization Source coupled with SCIEX 6500 triple-quadrupole mass spectrometer. The spectrum was recorded in a positive mode. The acidic peak in the mass spectrum of anti-arrhythmic drugs appeared as [M + H]^+^, and the highest peak of the fragment was chosen as the product ion. The formation of [M + H]^+^ species and the product ions were identical to that in the published literature^7^.

### Validation of DART–MS/MS method

#### Linearity

The linearity of the calibration curve was measured by preparing six concentration levels of analytes in serum. The analyte peak area/IS peak area ratio versus nominal analyte concentration (x) was calculated and the linear regression was performed using a weighting factor of 1/x^2^. The slope, intercept, and correlation coefficient were calculated for each calibration curve.

#### Selectivity and specificity

Selectivity was evaluated in human serum from six different individuals. Blank (drug-free) serum samples were compared with the serum plasma samples at the LLOQ. The peak areas in the blank samples should be ≤ 20% compared to the peak areas of the LLOQ samples in each batch. Cross analyte/IS interferences were determined by spiking IS into blank serum samples and the peak areas of the analytes should be ≤ 20% compared to the peak areas of the LLOQ.

#### Accuracy and precision

The accuracy and precision were determined by quality control samples at LLOQ, low, middle, and high concentrations. Six replicates of quality control (QC) samples at each level were prepared and analyzed in three different batches. The accuracy (intra- and inter-batch accuracy) was determined by the deviation percent between the calculated value and the nominal value. The precision was determined by calculating the coefficient of variance of six (intra-batch precision) or eighteen (inter-batch precision) values in four QC levels.

#### Recovery and matrix effect

Recovery and matrix effect was determined by preparing three QC samples (low, middle, and high) with blank serum from six different individuals. Recovery was evaluated by comparing the peak areas of analytes spiked before precipitation to the peak areas of analytes spiked after precipitation. Matrix effect was calculated by comparing the peak areas of the analytes spiked after precipitation to the peak areas of analytes in chemical solvent in the same levels. The IS-normalized matrix effect factor was calculated as the ratio between the absolute matrix effect factor of analytes and IS.

### Development and validation of LC–MS/MS method

The reference LC–MS/MS method was developed according to the previous report^7^ with slight modifications. The linear range and QC levels were the same as the DART–MS/MS method. An ExionLC system coupled with a SCIEX 4000 Triple Quad LC/MS (SCIEX, Framingham, MA, U.S.A.) was used for the LC–MS/MS analysis. A 10 μl aliquot of the precipitated supernatant was injected onto a Luna Omega C18 100A, 2.1 × 50 mm, 1.6 μm, Phenomenex column at the flow rate of 0.4 ml/min. The mobile phase A was 1 mM ammonium acetate and 0.1% formic acid in H_2_O and the mobile phase B was 1 mM ammonium acetate and 0.1% formic acid in acetonitrile. For gradient elution, 85% mobile phase A was used for the first 3.0 min, followed by 95% mobile phase B for 1.0 min, and then back to 85% mobile phase A for 2.0 min. The total sample acquisition period was 6.0 min per sample. The LC–MS/MS method was validated in terms of linearity, accuracy and precision, and sensitivity.

### Data processing and statistical analysis

The multiple injection mode was able to rapidly quantify multiple peaks in one chromatography automatically. Ten or twelve sample acquisitions were conducted in one acquisition period. The multiple injection mode of MultiQuant 3.03 program (SCIEX, Framingham, MA, USA) was applied to peak integration and data processing. For rapid quantification, the time of the first injection was set at 45 s and the interval was set at 15 s for each injection. Results were expressed as means ± standard error mean (SEM) of six replicates or three independent batches unless otherwise specified.

## Results

### Development and optimization of the DART–MS/MS method

**Figure 2:**
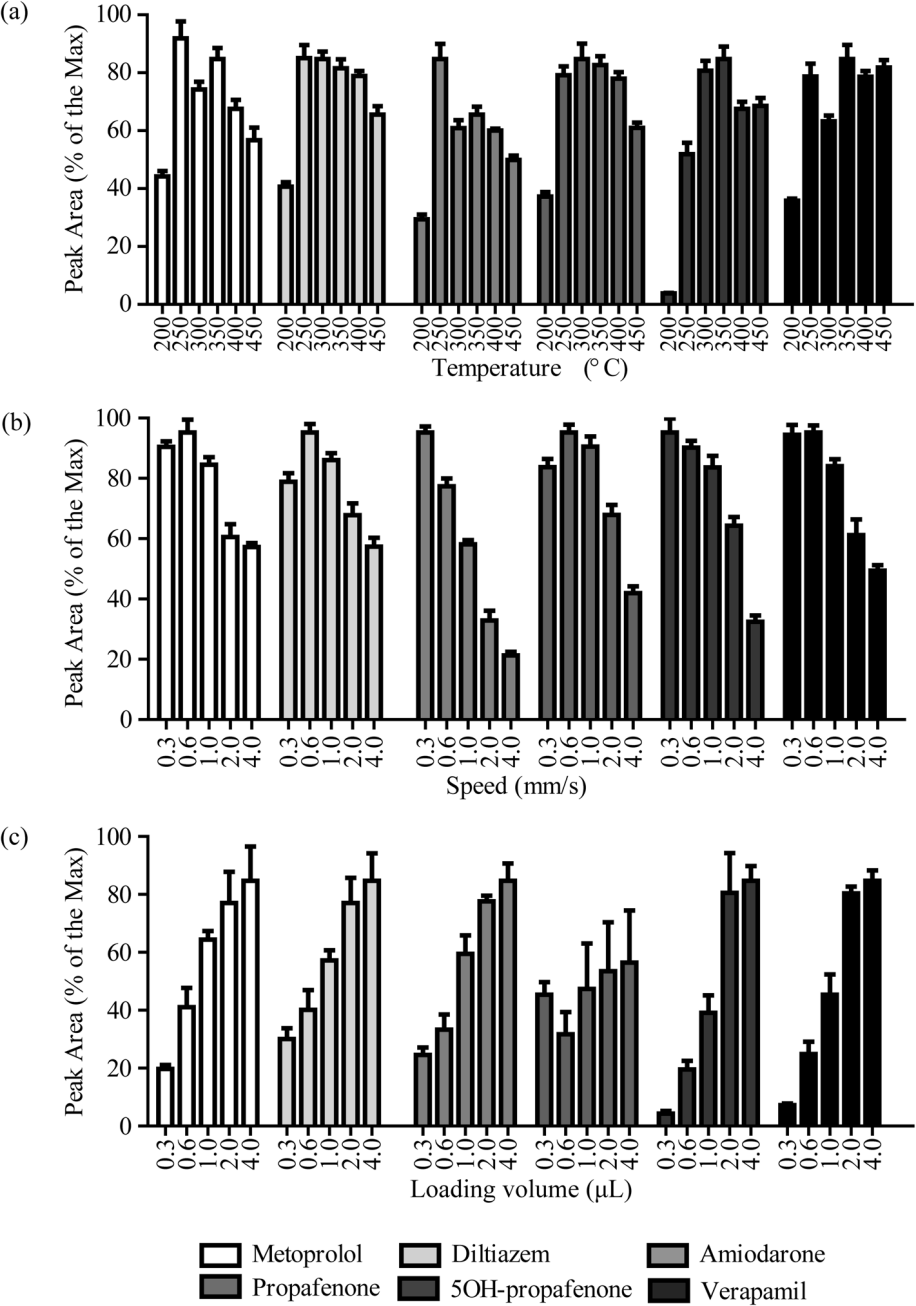
The helium gas temperature, linear rail speed, and sample volume affect the peak area of analytes. (**a**) Helium gas temperature; (**b**) linear rail speed; (**c**) sample volume, n = 6.

DART–MS/MS conditions were optimized in an attempt to achieve sharp and selective peaks with maximum intensity. Appropriate tuning parameters were utilized to determine the precursor and product ions of analytes and IS using ESI^+^ mode. The detailed precursor and product ions were listed in Table 2 and the collision energy (CE) was optimized for each analyte. We then carefully evaluated the temperature of DART helium gas, the moving speed of X rail, and the sample loading volume in this study. As shown in Fig. 2a, the peak areas of metoprolol, diltiazem, and amiodarone were highest at 250 °C. For propafenone, 5-hydroxy(OH)-propafenone, and verapamil, the peak areas at 250 °C were equivalent to those at 350 °C. Therefore, 250 °C was employed as the best condition. For the moving speed of the X rail, the peak area attained the highest value at 0.6 mm/s for metoprolol, diltiazem, propafenone and verapamil (Fig. 2b). For amiodarone and 5OH-propafenone, the peak area reached the high level at 0.3 mm/s and 0.6 mm/s and was sharply reduced at 1 mm/s, 2 mm/s and 4 mm/s, indicating that the high speed could suppress the desorption rate and ionization. Collectively, 0.6 mm/s was finally selected as the optimal condition. In terms of the sample loading volume, the peak area increased significantly from 0.3 μL to 2 μL, while increasing the sample volume from 2 μL to 4 μL did not generate a proportional increase in peak areas (Fig. 2c). Therefore, the loading volume of the samples was set at 2 μL for the analysis.

**Table 2:** Multiple reaction monitoring (MRM) transitions and collision energy of six anti-arrhythmic compounds and internal standards.

Compound	Precursor ion (m/z)	Product ion (m/z)	Collision energy (V)
Metoprolol	268.1	116.1	26
Metoprolol-d7	275.1	123.1	26
Diltiazem	415.2	178.0	33
Amiodarone	646.1	58.1	100
Amiodarone-d4	650.1	58.1	100
Propafenone	342.1	116.1	29
Propafenone-d5	347.1	121.1	29
5OH-propafenone	358.1	116.0	29
5OH-propafenone-d5	363.1	121.1	29
Verapamil	455.3	165.3	37

The stable-isotope-labeled analogs were utilized as internal standards of their original compounds including metoprolol-d7, amiodarone-d4, propafenone-d5, and 5OH-propafenone-d5. Besides, metoprolol-d7 and 5OH-propafenone-d5 was also served as an internal standard for diltiazem and verapamil, respectively.

### Validation of DART–MS/MS method

**Figure 3:**
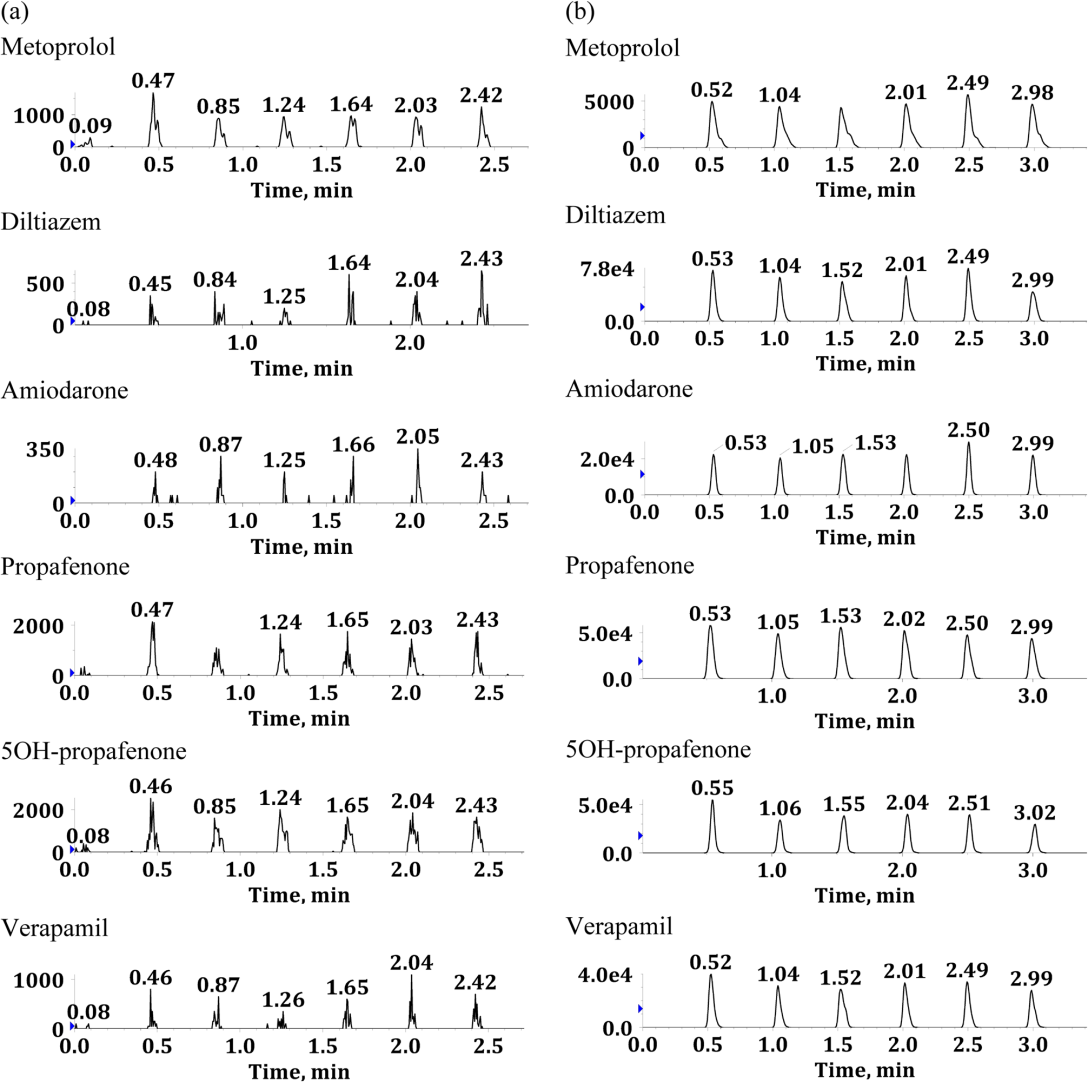
Representative chronograms of drug-free serum (**a**) and spiked with LLOQ level (**b**) in six different sources.

In the method validation, linearity, selectivity, specificity, accuracy, precision, recoveries, and matrix effect was assessed following US-FDA and EMA guidelines on bioanalytical method validation^25,26^. The calibration standards were fitted to the linear regression with good linearity and reproducibility in the calibration range (Table 3). Correlation coefficients (R^2^) were ≥ 0.9906, and the bio-analytical assay was linear over 400 fold for all analytes. As shown in Fig. 3, the peak areas of blank serum samples were < 20% of the peak areas of LLOQ samples from six different sources except metoprolol indicating good selectivity. Besides, there was a negligible crosstalk between MS channels of analytes and internal standards (see Supplementary Fig. S2 and S3 online).

**Table 3:** Linear range, coefficient of correlation (R^2^), and linear regression equation.

Analyte	Linear range (ng/mL)	R^2^	Linear regression equation
Metoprolol	10–4,000	0.9906	y = 0.000104406x + 0.00152
Diltiazem	10–4,000	0.9982	y = 0.02264x + 0.06713
Amiodarone	50–20,000	0.9950	y = 0.000189274x + 0.000515430
Propafenone	25–10,000	0.9937	y = 0.00883x + 0.04537
5OH-propafenone	25–10,000	0.9940	y = 0.00813x + 0.03170
Verapamil	10–4,000	0.9976	y = 0.01697x − 0.06363

The intra-run and inter-run accuracy and precision for the quantification of six anti-arrhythmic drugs in human serum samples at four QC levels are presented in Table 4. The intra-batch and inter-batch accuracy was in the range of 86.1–109.9% and 90.9–103.2%, respectively. The intra- and inter-batch precision were in the range of 2.9–11.8% and 6.3–14.3%, respectively. The results demonstrated acceptable bioanalytical assay accuracy and precision parameters. The recoveries of six anti-arrhythmic drugs and internal standards were ranged from 90.7 to 108.5% indicating reproducible recovery across the concentration range studied (Table 5). The matrix effect was evaluated by comparing the peak area ratio of spiked samples post precipitation versus the chemical QCs. The data showed that the matrix effect was in the range of 89.4–105.6% (Table 5). The IS-normalized matrix effect was 91.2 ± 3.9%, 93.3 ± 7.3%, 98.8 ± 3.1%, 98.4 ± 5.7%, 97.6 ± 6.3%, 94.4 ± 4.5% for metoprolol, diltiazem, amiodarone, propafenone, 5-hydroxy-propafenone and verapamil, respectively. Therefore, the matrix did not affect the sensitivity or selectivity of the present method.

**Table 4:** Accuracy and precision of anti-arrhythmic compounds in human serum.

Analyte	Intra-batch accuracy (precision) n = 6				Inter-batch accuracy (precision) n = 18			
	LLOQ	Low	Middle	High	LLOQ	Low	Middle	High
Metoprolol	101.2 (11.8)	106.7 (9.5)	99.9 (6.8)	101.2 (9.5)	103.0 (7.6)	102.4 (8.9)	98.8 (8.6)	97.3 (9.3)
Diltiazem	106.1 (5.3)	90.3 (7.3)	95.7 (7.2)	86.1 (10.9)	102.9 (9.8)	95.8 (9.7)	99.3 (9.6)	90.9 (14.3)
Amiodarone	103.4 (5.0)	101.0 (5.5)	94.0 (4.9)	94.4 (4.1)	100.8 (10.4)	102.6 (7.8)	95.2 (8.2)	93.5 (6.6)
Propafenone	109.9 (7.9)	98.6 (5.8)	96.8 (9.8)	86.4 (2.9)	101.0 (12.0)	101.0 (6.3)	98.5 (9.0)	95.7 (9.6)
5OH-propafenone	102.0 (11.7)	98.8 (8.3)	96.7 (7.0)	94.5 (5.2)	99.6 (14.0)	102.6 (9.3)	95.9 (7.2)	99.2 (6.7)
Verapamil	106.7 (11.5)	98.0 (5.9)	95.3 (6.0)	102.4 (8.7)	103.2 (12.2)	101.4 (9.7)	95.2 (7.1)	92.8 (10.8)

**Table 5:** Recovery and matrix effect of anti-arrhythmic compounds and internal standards in human serum.

Analyte	Recovery%	Matrix effect%
Metoprolol	97.5 ± 5.6	96.7 ± 5.0
Metoprolol-d7	95.8 ± 5.7	105.6 ± 4.3
Diltiazem	94.3 ± 5.6	102.4 ± 9.6
Amiodarone	90.7 ± 8.4	94.4 ± 2.3
Amiodarone-d4	99.2 ± 8.2	98.5 ± 5.2
Propafenone	93.0 ± 5.9	92.7 ± 4.3
Propafenone-d5	108.5 ± 5.0	95.2 ± 8.1
5-Hydroxy-propafenone	92.1 ± 6.3	88.9 ± 5.9
5-Hydroxy-propafenone-d5	98.0 ± 5.8	97.8 ± 7.3
Verapamil	93.8 ± 5.8	89.4 ± 4.2

### Analysis of clinical TDM samples with DART–MS/MS method

Thirty clinical TDM samples of arrhythmic drugs were collected from January to November of 2018 after 4 h of administration of anti-arrhythmic drugs. The dosage of metoprolol, diltiazem, amiodarone, propafenone, and verapamil were 100 mg, 420 mg, 400 mg, 300 mg, and 120 mg, respectively. There were six samples for each analyte and the clinical TDM samples were pre-treated and analyzed with the validated DART–MS/MS method. The total acquisition time of DART–MS/MS was less than 18 min. The correlation coefficients (R) of six analytes were ≥ 0.9903. The accuracy of quality control samples was in the range of 89.2–113.8%. The detailed results of clinical sample concentrations were listed in Table 6.

**Table 6:** The concentration of anti-arrhythmic compounds in 30 serum samples by DART–MS/MS and LC–MS/MS.

Analyte	DART–MS/MS (ng/mL)	LC–MS/MS (ng/mL)	RE (%)	Analyte	DART–MS/MS (ng/mL)	LC–MS/MS (ng/mL)	RE (%)
Metoprolol	818.6	754.0	8.6	Propafenone	1887.5	1,710.0	10.4
	803.8	774.0	3.9		1,019.3	1,020.0	− 0.1
	1,421.3	1,330.0	6.9		1,620.7	1,830.0	− 11.4
	133.0	126.0	5.6		216.0	198.0	9.1
	265.8	259.0	2.6		458.7	513.0	− 10.6
	404.5	374.0	8.2		111.8	102.0	9.6
Diltiazem	760.3	804.0	− 5.4	5OH-propafenone	1910.0	1,890.0	1.1
	1,181.2	1,270.0	− 7.0		1,069.4	1,040.0	2.8
	628.0	618.0	1.6		1950.2	1,850.0	5.4
	119.0	109.0	9.2		261.2	240.0	8.8
	742.0	712.0	4.2		660.2	599.0	10.2
	561.0	511.0	9.8		126.4	123.0	2.8
Amiodarone	2,664.0	2,970.0	− 10.3	Verapamil	919.1	1,040.0	− 11.6
	507.5	497.0	2.1		663.8	673.0	− 1.4
	3,864.6	4,430.0	− 12.8		66.1	69.7	− 5.2
	389.0	370.0	5.1		1,406.8	1,320.0	6.6
	1635.2	1,580.0	3.5		218.7	223.0	− 1.9
	298.9	294.0	1.7		562.5	524.0	7.3

### Validation of the LC–MS/MS method

To verify the results of the clinical TDM samples obtained by the DART–MS/MS method, an LC–MS/MS method was developed and validated. The validation results were shown in the supplementary information (see Supplementary Table S1 online). The same series of calibration standards and QC samples were employed. The linear regression was conducted and the coefficient of correlation (R^2^) was ≥ 0.9936, which was similar to that of the DART–MS/MS method. The intra- and inter-batch accuracy were ranged from 95.6 to 104.9% and the intra- and inter-batch precision was within 9%. Therefore, the LC–MS/MS method was adopted as a reference method for evaluating the accuracy of DART–MS/MS in clinical samples.

### Comparison of DART–MS/MS and LC–MS/MS results

**Figure 4:**
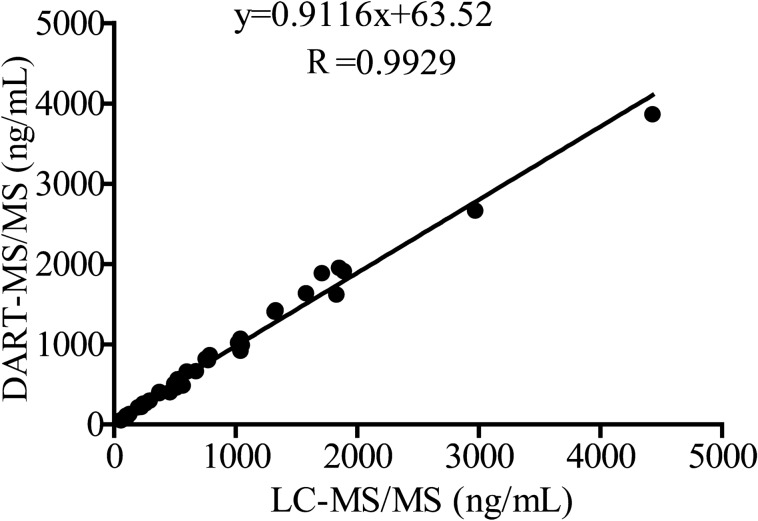
Correlation plot between concentrations measured by LC–MS/MS and DART–MS/MS for five anti-arrhythmic drugs and one metabolite in human serum, n = 30.

Thirty clinical TDM samples of arrhythmic drugs were then re-analyzed by the validated LC–MS/MS method. The total analysis time is more than 3.5 h. The results were listed in Table 6. The relative error (RE) of the concentrations obtained by LC–MS/MS and DART–MS/MS was within ± 13%. The back-calculated levels of the DART–MS/MS method and LC–MS/MS were then plotted to fit in a linear regression model. The slope and the coefficient of linearity were calculated. As shown in Fig. 4, the correlation plot of DART–MS/MS concentrations (Y-axis) versus those of LC–MS/MS (X-axis) was provided. The results showed that the values fitted a linear model with a coefficient (R) of 0.9929 and a slope of 0.9116. Besides, the values were paired and analyzed with the Student t-test. The results of DART–MS/MS and LC–MS/MS provided equivalent values for anti-arrhythmic drugs at the 95% confidence level. Therefore, DART–MS/MS had similar accuracy and precision with that of LC–MS/MS but took less time for clinical sample analysis.

## Discussion

To date, the studies aim for clinical quantitative analysis with DART are rare^22,25^. In our opinion, the reproducibility and throughput are the major concerns against the wide application of DART to quantitative analysis. Since the desorption efficiency of the heat helium gas is affected by many factors such as temperature, angle, time, and position^21^, ambient ionization techniques are more vulnerable by the fluctuation of the signal. In this method, the repetitiveness of the experiment was improved by adding the stable isotope-labeled internal standard (SIL-IS) in the sample preparation. Four analytes (metoprolol, amiodarone, propafenone, and 5OH-propafenone) were calibrated by their corresponding SIL-IS. Diltiazem and verapamil utilized metoprolol-d7 and 5OH-propafenone-d5 as internal standard because they had similar matrix effect and extraction recoveries. Since the similarity of chemical structure and properties, SIL-IS was able to offset the fluctuations of sample loading volumes and the desorption rate at different positions. Besides, the addition of SIL-IS was adopted by multiple reported methods of ambient ionization for improving reproducibility^17,27^. Therefore, SIL-IS is very needed for the correction of these factors and reduce the fluctuation of signals especially for the simultaneous determination of multiple analytes.

Most of the previous DART methods utilizes 10 or 12 position loading system, which requires frequent sample loading for clinical samples in large quantities^28–30^. In this study, an XZ transmission module and QuickStrip 96 sample card were applied to increase the assay throughput. For the first time of our knowledge, Direct Analysis in Real Time (DART) was proposed as a rapid, high throughput and quantitative tool for the simultaneous determination of five arrhythmic drugs and one active metabolite in human serum. Without the time-consuming chromatographic separation and maintenance of the related equipment of chromatography system, the DART–MS/MS method has short cycle time (< 30 s per sample), large throughput (96 samples), and small sample volume (2 μL). Rapid analysis techniques including laser desorption/ionization techniques and paper spray have also been explored for TDM analysis^31^. However, these methods may be a lack of accuracy and reproducibility in the analysis of clinical samples. The DART–MS/MS method in this study was proven to be rapid, high-throughput, accurate, and precise.

Therapeutic drug monitoring prevents the drug concentration-dependent adverse reactions (ADR) and monitors the compliance. The concentrations of clinical TDM samples above the upper limits may have increased risks of ADRs^32,33^. The reported effective plasma concentration range was from 40 to over 3,000 ng/mL for propafenone and 500 to 2,500 ng/mL for amiodarone^32^. Also, the patients’ compliance should be monitored for β-blockers and calcium-channel blockers^5^. In this study, the median values of thirty TDM samples concurred with previous observations with similar dosages^3,8,34–36^. The concentrations over or below the ranges reported indicated the possibility of overdose or missing dose. Therefore, this study is a proof-of-concept in the use of DART–MS/MS for the absolute quantification of drugs in TDM. DART–MS/MS method will be of high value in attempting the quantitative TDM analysis of other drugs such as tacrolimus, methotrexate, etc.

## Conclusion

We developed a DART–MS/MS method for absolute and simultaneous quantitative analysis of metoprolol, diltiazem, amiodarone, propafenone, 5-hydroxy(OH)-propafenone, verapamil in human serum. The method involves a short running time (30 s per sample) and gives high sensitivity using only 2 μL of samples. The isotope-labeled internal standard is added during sample preparation. The method is validated in terms of linearity, selectivity, accuracy, precision, recoveries, and matrix effect. The DART–MS/MS method is applied in daily analysis of clinical TDM samples with acceptable accuracy. The present method is proposed to be of value in TDM for patients on the therapy of anti-arrhythmic drugs for monitoring toxicity and compliance. Besides, this study proves that DART–MS/MS is of high value in rapid and quantitative analysis in biological matrices without chromatographic separation.

## Supplementary information

Supplementary information.
