# Supplementary material for: Direct analysis in real time-mass spectrometry for rapid quantification of five anti-arrhythmic drugs in human serum: application to therapeutic drug monitoring
Source: Sci Rep. 2020 Sep 23;10:15550. doi: 10.1038/s41598-020-72490-w (PMC7511339; doi:10.1038/s41598-020-72490-w)
Supplement: Supplementary file 1 — Supplementary information. [file 41598_2020_72490_MOESM1_ESM.docx]

**Supplementary information**

Direct analysis in real time-mass spectrometry for rapid quantification of five anti-arrhythmic drugs in human serum: application to therapeutic drug monitoring

Yuzhou Gui^a,b,c^, Youli Lu^a,c^, Shuijun Li^a,c^, Mengqi Zhang ^a,c^, Xiaokun Duan^d^, Charles C. Liu^d^, Jingying Jia^a,c*^, Gangyi Liu^a,c*^

^a^ Central Laboratory, Shanghai Xuhui Central Hospital, Shanghai, 200031, China

^b^ Zhongshan-Xuhui Hospital, Fudan University, Shanghai, 200031, China

^c^ Shanghai Engineering Research Center of Phase I Clinical Research & Quality Consistency Evaluation for Drugs, Shanghai, 200031, China

^d^ ASPEC Technologies Limited, Beijing, 100101, China

**Name and address for correspondence**

Gangyi Liu, Jingying Jia

Central Laboratory, Shanghai Xuhui Central Hospital, Shanghai Engineering Research Center of Phase I Clinical Research & Quality Consistency Evaluation for Drugs, 966 Middle Huaihai Road, Shanghai, 200031, People’s Republic of China.

Tel./Fax: +86-21-54030254;

E-mail address: [gyliu@shxh-centerlab.com](mailto:gyliu@shxh-centerlab.com); [jyjia@shxh-centerlab.com](mailto:jyjia@shxh-centerlab.com)

**Supplementary Figure S1.** Chemical structure of anti-arrhythmic compounds and internal standards. (a) metoprolol (b) diltiazem (c) amiodarone (d) propafenone (e) 5-hydroxy-propafenone (f) verapamil (g) metoprolol-d7 (h) amiodarone-d4 (i) propafenone-d5 (j) 5-hydroxy-propafenone-d5


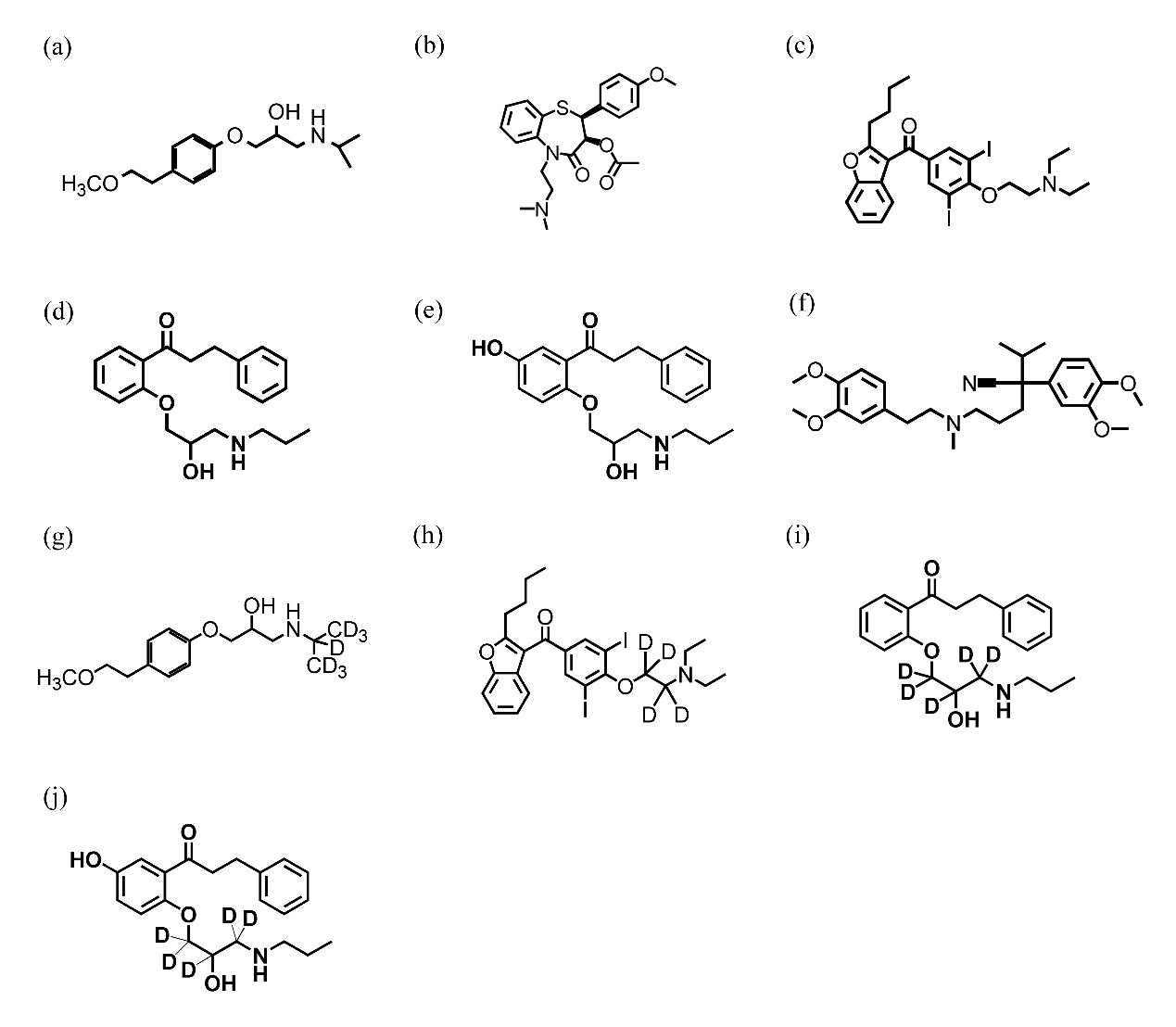


**Supplementary Figure S2.** Typical chronograms of drug-free serum spiked with internal standards (a) metoprolol (b) diltiazem (c) amiodarone (d) propafenone (e) 5-hydroxy-propafenone (f) verapamil


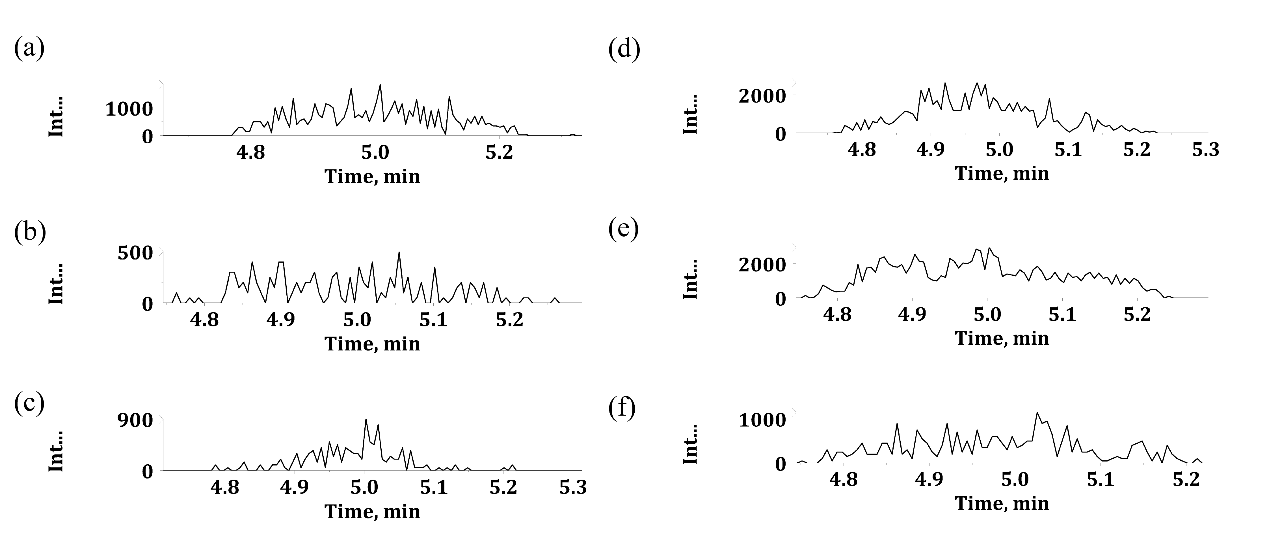


**Supplementary Figure S3.** Typical chronograms of drug-free serum spiked with internal standards (Black) metoprolol-d7 (Purple) amiodarone-d4 (Red) propafenone-d5 (Green) 5-hydroxy-propafenone-d5

**
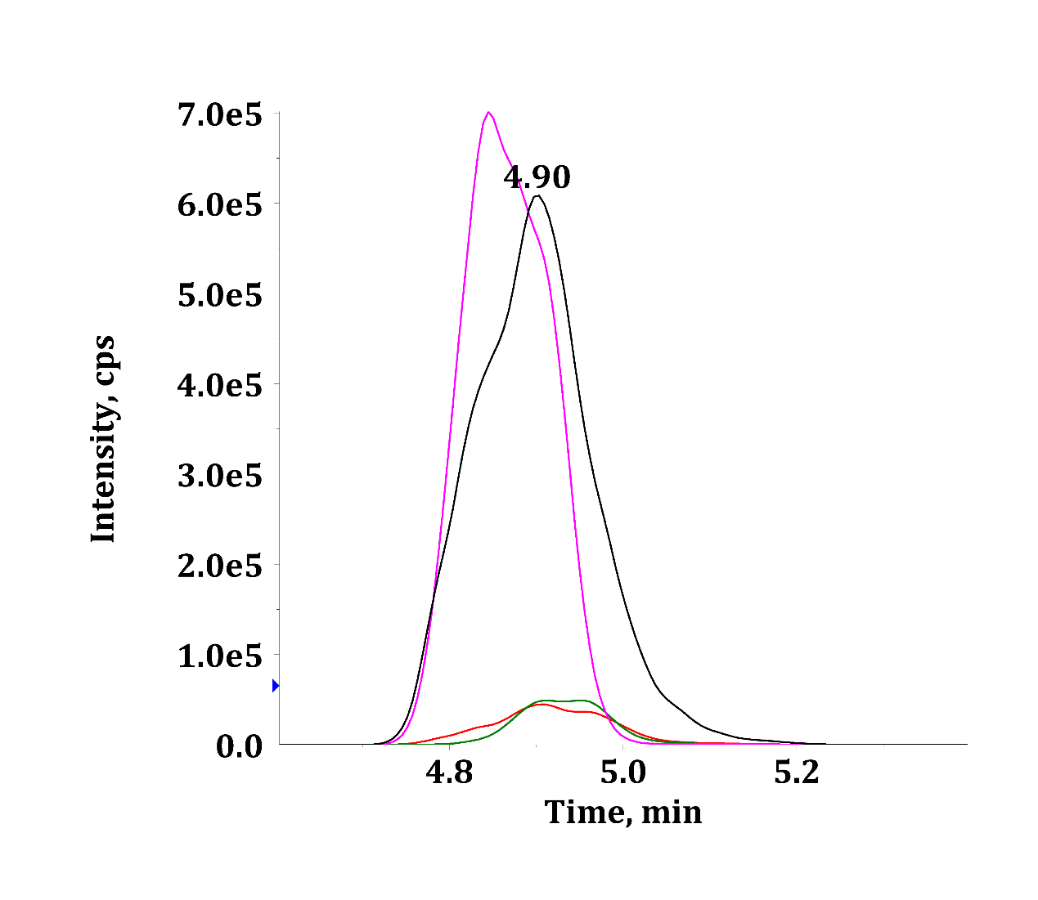
**

Supplementary Table S1. The correlation coefficient, accuracy and precision of anti-arrhythmic drugs in human serum by LC-MS/MS.

| Analyte | Correlation Coefficient  (R^2^) | Intra-batch accuracy (precision), n=6 | | | | | Inter-batch accuracy (precision), n=18 | | | |
| --- | --- | --- | --- | --- | --- | --- | --- | --- | --- | --- |
|  |  | LLOQ | Low | Middle | High | LLOQ | | Low | Middle | High |
| Metoprolol | 0.9936 | 104.5 (2.1) | 99.0 (4.3) | 96.4 (3.9) | 97.9 (6.3) | 103.8 (0.1) | | 99.0 (0.1) | 98.4 (3.5) | 97.8 (0.6) |
| Diltiazem | 0.9984 | 97.8 (5.6) | 100.6 (4.4) | 100.7 (4.9) | 99.0 (8.1) | 98.8 (3.0) | | 99.0 (1.7) | 102.3 (1.5) | 100.5 (3.2) |
| Amiodarone | 0.9994 | 100.7 (2.3) | 104.6 (4.0) | 100.3 (5.3) | 100.7 (4.0) | 100.2 (2.3) | | 102.3 (3.0) | 100.1 (2.5) | 100.0 (1.4) |
| Propafenone | 0.9974 | 104.9 (3.5) | 104.0 (4.9) | 102.0 (4.1) | 100.6 (4.9) | 104.4 (1.91) | | 101.3 (2.5) | 100.9 (2.1) | 99.4 (1.2) |
| 5OH-propafenone | 0.9976 | 102.2 (4.1) | 98.9 (6.6) | 102.4 (3.9) | 98.4 (6.4) | 99.5 (4.62) | | 99.2 (0.6) | 101.4 (1.8) | 100.0 (2.8) |
| Verapamil | 0.9988 | 104.0 (2.3) | 100.2 (3.6) | 95.6 (4.2) | 98.3 (2.5) | 102.8 (1.02) | | 99.3 (0.8) | 100.1 (3.9) | 98.3 (0.1) |
